# From plastics to microplastics and organisms

**DOI:** 10.1002/2211-5463.13120

**Published:** 2021-04-01

**Authors:** Oliver Bajt

**Affiliations:** ^1^ Marine Biology Station National Institute of Biology Piran Slovenia; ^2^ Faculty of Maritime Studies and Transport University of Ljubljana Slovenia

**Keywords:** degradation, fish, marine environment, microplastics, mussels

## Abstract

The amount of plastic waste and microplastics released into marine environments has increased rapidly in recent decades. The durability of plastic materials results in major problems following their release into the environment. This study provides an overview of recent findings on issues related to plastic degradation, the accumulation of microplastics in mussels and fishes, and the toxicological effects associated with the ingestion of microplastics. These findings confirm the serious problem of slowly degrading plastics (which rarely degrade fully) in natural marine environments. Microplastics have become widespread pollutants and have been detected in mussels and fish around the world. Microplastic particles, whether virgin or with adsorbed pollutants on their surfaces, pose a health problem after being ingested by marine organisms. This paper ends by highlighting the need for certain improvements in studies of these phenomena.

## Graphical abstract

This study provides an overview of recent findings on the problem of microplastics in the marine environment. Microplastic pollution is a very widespread problem. Microplastics have been identified in different mussel and fish species. Many studies also emphasize the adverse toxic effects on marine organisms after the ingestion of microplastics.

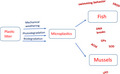

**Abbreviations**
FTIR: Fourier transform infrared
HDPE: high‐density polyethylene
LDPE: low‐density polyethylene
PA: polyamide
PE: polyethylene
PET: polyethylene terephthalate
PP: polypropylene
PS: polystyrene
PVC: polyvinyl chloride

## Introduction

Plastic products are being produced at ever‐increasing rates, making plastic pollution one of the most important contemporary environmental issues. Plastic is a very practical material that is long‐lasting, resistant to degradation, inert, and easy to shape, with very low production costs. It has therefore become an important material for everyday use (Table 1). Annual global production of plastic materials is about 300 million tons, increasing more than 20‐fold in the last 60 years. In Europe, only about 30% of plastic material is recycled. About 10% of the plastic produced each year ends up in the ocean and about 8 million pieces of plastic escape into the oceans from land‐based sources every day, mostly by rivers (https://plastic‐pollution.org/). Once at sea, plastic material can be caught up in ocean currents and transported to the open sea. Plastic material is very resistant in the natural environment and requires centuries to decompose. The biodegradation of plastics is a very slow process, since few naturally occurring microorganisms recognize these man‐made materials. Sunlight, wind, and waves continuously break down plastic into smaller and smaller particles. Although the photodegradation process can proceed down to the molecular level, the degradation products remain polymers and may contribute to the dissolved organic carbon pool in the ocean [1, 2, 3].

**Table 1:** Main plastics and their applications.

Polymer	Abbreviation	Use
Polyethylene terephthalate	PET	Containers/bottles for beverages (juice, water, beer), detergents, butter jars, plastic film, microwavable packaging
High‐density polyethylene	HDPE	Opaque milk, water, and juice containers, detergent and shampoo bottles, garbage bags, yogurt and margarine tubs, molded plastic cases
Low‐density polyethylene	LDPE	Bread and frozen food bags, most plastic wraps, and squeezable bottles (honey, mustard), outdoor furniture, floor tiles, shower curtains
Polypropylene	PP	Bottle caps, ketchup bottles, yogurt and margarine containers, medicine and syrup bottles, drinking straws, opaque plastic containers, including baby bottles, plastic pressure pipe system
Polyvinyl chloride	PVC	Toys, clear food and nonfood packaging (e.g., cling wrap), some squeeze bottles, shampoo bottles, cooking oil and butter jars, detergent and window cleaner bottles, shower curtains, medical tubing, and numerous construction products, electrical cable/wire insulation
Polystyrene	PS	Food containers, egg cartons, disposable cups, plates, cutlery, plastic tableware, take‐out food containers, plastic cutlery, compact disk cases
Polyamide	PA	Fibers, fishing line, toothbrush bristles, tubing
Polycarbonate	PC	Compact disks, eyeglasses, security windows, traffic lights and lenses

Plastics often contain additives to enhance their mechanical properties, flexibility, durability, stability, and color. The weathering and degradation of plastic materials can lead to the leaching of these compounds into the seawater and their ingestion by organisms [4, 5].

Microplastics are pieces of plastic measuring < 5 mm in length which are divided into primary and secondary microplastics. Primary microplastics may be found in personal care products (microbeads) or in the form of plastic pellets used in industrial manufacturing or plastic fibers used in synthetic textiles. These particles directly enter natural ecosystems from different sources. Secondary microplastics are broken down from larger particles through natural weathering processes. Both types of microplastics have been found to accumulate and persist in natural aquatic ecosystems.

Microplastic particles can be harmful to marine life. Ingested plastic fragments can cause changes in feeding and reproductive behavior as well as increased mortality. Their toxic effect is mostly due to the release of different harmful compounds from the plastic material and pollutants adsorbed on the surface of floating particles [4]. Numerous studies show the harmful effects and bioaccumulation among different groups of marine organisms including fish, bivalves, crabs, seabirds, phytoplankton, corals, and meiofauna. Plastic surfaces are also good substrates for the adsorption of different non‐native species and their transport to remote areas around the world.

This paper presents the results of recent studies on the degradation of plastic materials into microplastics. It further highlights the importance of this rapidly growing problem by providing an overview of recent findings on the distribution and effects of microplastics on mussels and fish and their importance for human nutrition.

**Table 2:** Microplastics in different mussel species from different regions worldwide.

Species	Area	Identified polymers	Shape	MP size (mm, average or range)	Average quantity (item/individual)	Average quantity (item/g w.w.)	Reference
*Aulacomya atra*	Patagonia, Argentina		Fiber	1.25		0.3	[32]
*Limnoperna fortunei*	Río de la Plata estuary, Argentina		Fiber	0.5–1.0	0.43	2.08	[33]
*Mytilus Galloprovincialis*	Adriatic Sea	PE, PP, PET, PS, PLY, PVC	Fiber, fragment	0.02–0.3		0.24–1.33	[27]
*Mytilus edulis*	French Atlantic coast	PE, PP	Fragment	0.05–0.1	0.61	0.6	[24]
*Crassostrea gigas*	French Atlantic coast	PE, PP, ABS, Polyester, PS	Fragment	0.05–0.1	2.1	0.18	[24]
*Mytilus galloprovincialis*	N Ionian Sea	PE, PP, PTFE	Fragment, fiber	0.04–0.74	1.9	2.5–5.3	[31]
*Perna viridis*	Fishing Harbour of Chennai, India	PS	Fiber, fragment	0.005–0.025			[34]
*Mytilus Galloprovincialis*	Italy		Filament (fiber)	0.75–6.0	3.0–12.4	4.4–11.4	[29]
*Mytilus Galloprovincialis*	Bizerte lagoon, Tunisia	PE, PP, celophane	Fiber, fragment		7.7	2.1	[28]
*Mytilus* spp.	Norwegian coastal waters	Celophane, EVA, PET, PP, PE, PA	Fiber, fragment	0.07–3.87	1.5	0.97	[26]
*Mytilus galloprovincialis*, *Choromytilus meridionalis, Aulacomya ater*	Cape Town, South Africa		Filament (fiber), Fragment, sphere	0.05–1.0	4.27	2.33	[67]
*Mytilus Galloprovincialis*	Turkish coasts	PET, PP, PE	Fragment, fiber, film	0.5–2.0	0.23	0.69	[30]
*Mytilus edulis*	Coastal waters, United Kingdom	Polyester, PP, PE	Fiber, fragment	0.07–4.7	1.1–6.4	0.7–2.9	[25]
*Mytilus edulis, Perna viridis*	Coastal waters China	PET, PVC, PE, PP, rayon	Fiber, fragment, bead	0.25–1.0	0.77–8.22	1.52–5.36	[35]
*Mytilus* spp.	North coast Spain		Fiber, fragment, pellet	0.2–1.0	2.19–2.81	1.59–2.55	[68]
*Mytilus edulis*	SW England	Modified cellulose, polyester, nylon	Fiber, fragment	0.3–1.3	1.43–7.64		[69]
*Crassostrea gigas, Crassostrea angulate, Crassostrea hongkongensis and Crassostrea sikamea*	China coastline	Celophane, PE, PET, PP, PA, PS, PC, PVC	Fiber, fragment, film	0.02–4.8	2.93	0.62	[70]
*Mytilus* spp., *Modiolus modiolus*	Scotland	Polyester, PET, poly(ether–urethane)	Fiber	0.2–2.0	3.2–3.5	0.086–3.0	[71]

**Table 3:** Microplastics in different fish species from different regions worldwide.

Species	Area	Identified polymers	Shape	MP size (mm, average or range)	Average quantity (item/individual)	Reference
*Solea solea*	Adriatic Sea	PVC, PP, PE, PA, polyester	Fragment, fiber	0.1–0.5	1.64–1.73	[40]
*Sardinia pilchardus*	Adriatic Sea	PP, PVC, PTFE, PA	Fragment, fiber	0.12–0.59	1.4–7.9	[38]
*Engraulis encrasicolus*	Adriatic Sea	PVC, PET	Fiber, fragment	0.81–1.86	0.5–2.8	[38]
*Triglops nybelini*	NE Greenland	Polyester, acrylate, PA, PE	Fiber	1.4	< 1	[53]
*Boreogadus saida*	NE Greenland	Polyester, acrylate, PA, PE	Fiber	1.8	1.1	[53]
Twenty–nine fish species	Bohai Sea, China	Cellophane, PET, PP	Fiber, fragment, film, pellet	0.02–5.0	2.14	[48]
*Boops boops*	Mediterranean Sea	PE, PP, PS, PVC	Filament (fiber)	0.05–4.75	1.17	[42]
Six fish species	Biobio, Chile	Polyester, PE, PET	Fiber	0.18–2.84		[51]
Nine fish species	W Arabian Gulf	PP, PE, LDPE	Fiber, fishing thread, fragment	0.66–3.55	0.057	[72]
Fourteen deep‐sea fish species	S China Sea	Cellophane, PA, PET	Film, fiber, granule	< 1–5	1.96	[47]
Six fish species	Haizhou Bay, China	Cellophane, PP, PE	Fiber, fragment, sheet	0.03–2	3.0–6.3	[49]
*Mugil cephalus*	E Hong Kong	PP, PE, polyester	Fiber, fragment	1.21	4.3	[50]
*Boops boops*	W Mediterranean Sea	PE, PP, PS	Fiber, fragment		1.8	[41]
*Sardina pilchardus,*	Gulf of Lyon, France	PET, PE, PA, PP	Fiber	1.77	0.20	[39]
*Engraulis encrasicolus*	Gulf of Lyon, France	PET, PE	Fiber	1.81	0.11	[39]
*Mullus barbatus*	Mediterranean Sea		Fiber, fragment, sheet	0.1–5.0	1.08	[36]
*Merluccius merluccius*	Mediterranean Sea		Fiber, fragment	0.1–2.5	1.38	[36]
*Sardina pilchardus*	N Ionian Sea	PE, PP	Fragment, fiber	0.04–0.86	0.8	[31]
*Pagellus erythrinus*	N Ionian Sea	PE, PP	Fragment, fiber	0.03–1.27	0.8	[31]
*Mullus barbatus*	N Ionian Sea	PE, PP, PET, PS	Fragment, fiber	0.04–0.8	0.5	[31]
Seven fish species	NW Atlantic	Methyl cellulose, PE, nylon	Fiber, fragment	0.04–8.2	1.15–2.36	[45]
Six fish species (open ocean and deep sea)	NE Atlantic	PE, PP, polyester	Fragment, fiber	0.02–5	0.04–0.22	[46]
*Dicentrarchus labrax*, *Diplodus vulgaris*, *Platichthys flesus*	Mondego estuary (Portugal)	Polyester, PP, polyacrylonitrile, PE, PA	Fiber, fragment	< 1–5	0.18–3.14	[44]
*Boops boops*	Catalan coast, Spain	PP, PE, PS	Fiber, fragment	< 0.1–5	0.50–1.68	[43]
*Acanthopagrus australis, Mugil cephalus, Gerres subfasciatus*	Sydney, Australia	Acrylic polyester, polyester, PP, rayon	Fiber, fragment		0.2–4.6	[52]

More than 200 recent papers were downloaded from Scopus and WoS databases and examined. In case of duplicate records, one was removed. The number of papers is increasing rapidly and we had to select papers to be included in this review (Tables 2 and 3).

## Degradation of plastic material to microplastics

Plastics are very resistant to degradation under natural conditions. The degradation of plastics in marine environments takes several hundreds of years. These processes are driven by a combination of sunlight, air (oxygen), heat, and moisture. Photodegradation is a process by which a compound is transformed by irradiation and the absorption of photons. Degradation leads to changes in color, physical properties, surface characteristics, formation of visible defects such as cracks, and the breaking of plastic material into smaller particles that leads to the formation of microplastics. Exposure to sunlight, especially to ultraviolet light, seems to be the most important driver of plastic degradation. The absorption of light with adequate energy (shorter wavelengths) cleaves the chemical bond, causing physical and chemical changes (oxidation) and resulting in fragmentation of polymeric material. Plastic degradation can also lead to the formation of very small pieces of plastic or even dissolved polymeric compounds that are bioavailable to microorganisms. Although these organisms can metabolize the polymeric compounds into CO_2_ [6], it is a very slow process.

Luo *et al*. [7] found many changes in the characteristics of microplastics during a simulated aging treatment. These included increased carbonyl content, increased specific surface area, and color change. Surface fragmentation and cracks allowed light and oxygen to reach internal surfaces and further oxidize the microplastics. Another consequence was the increased release of pigments. Different polymers exhibited contrasting degradation rates in natural marine environments [8]. While polyvinyl chloride (PVC) released estrogenic compounds in seawater quite rapidly, polyethylene terephthalate (PET) underwent small surface changes with no estrogenic activity detected. Meanwhile, poly(butylene adipate‐co‐terephthalate) (PBAT), a copolymer, exhibited a heterogeneous surface with some cavities after weathering. When exposed to a marine environment, acrylonitrile butadiene styrene (ABS) became smoother and discolored with a deformed, fractured, and fragmented structure and a fouling of the plastic surface and leaching of additives and pigments from the matrix [9]. As plastic particles become weathered in the marine environment, their molar mass has been found to decrease, revealing the degradation of the polymer chain [10].

Artificial photodegradation has also been shown to lead to an increase in VOCs including carbonyls, lactones, esters, acids, alcohols, ethers, and aromatics [11].

A study of the effects of artificial seawater on polyethylene (PE)‐based beads showed significant structural and morphological changes [12]. Artificial seawater induced severe microcracking of the pellets' surfaces, the formation of oxidized groups, alterations in the thermal stability of the PE pellets, and an increase in the organic matter content of the water in which the pellets were kept.

The degradation of different plastic materials resulted in a measurable increase in the release of particles into the surrounding solution, with as many as a few million particles found per milliliter after 112 days of degradation [13]. A study of artificial photodegradation and fragmentation of PE films in air and water found that fragmentation only occurred in water, although air weathering did result in higher levels of oxidation [14]. Furthermore, after 25 weeks of weathering in water, 90% of the fragments were under 1mm in size, with very similar shapes, while micrometric fragments were not yet abundant. Therefore, weathering in water requires longer timeframes and multiple steps of fragmentation before microparticles are produced.

The photoaging of polystyrene (PS) microparticles under simulated sunlight irradiation has been associated with the formation of reactive oxygen species such as singlet oxygen and hydrogen peroxide, which are highly reactive with PS microparticles [15]. Long‐term photoaging led to a greater number of oxidative functional groups and higher negative charges on particle surfaces, resulting in stronger electrostatic repulsion and slower sedimentation in water. Different polymeric materials exhibit different degradation patterns. A study examining the accelerated weathering of high‐density polyethylene (HDPE), high impact polystyrene (HIPS), nylon 6, and polypropylene (PP) under UV light in simulated seawater found that HDPE and nylon 6 broke down into microfibers, while HIPS and PP did not physically degrade [16]. Pellets of PP, PE, and PS exposed to UV light in air and ultrapure and simulated seawater were all found to have increased numbers of oxidized functional groups [17]. The reaction medium was shown to affect rates of photochemical weathering, though crack and flake formation was a common feature of weathering in all media studied.

Biodegradation is a natural process that breaks down or changes the structure of organic compounds by the action of microorganisms. It depends on different exposure conditions (temperature, moisture, pH, nutrient availability), substrate characteristics (molecular weight, morphology, functional groups, crystallinity, cross‐linking), and the microorganisms involved (extracellular enzymes, hydrophobicity, number of microorganisms). Plastics are not readily degradable and its biodegradation in natural water bodies takes several years. The process by which plastics biodegrade in the marine environment is very complex. The first step is the formation of a microbial biofilm on the polymer surface, followed by deterioration and the fragmentation of the polymers into oligomers, dimers, and monomers by enzymatic activity. The final step is the mineralization to CO_2_ and H_2_O [18].

A microcosm experiment [19] in which a mixture of naturally weathered plastic pieces was incubated with an indigenous pelagic community resulted in an increase in the fraction of double bonds in the surface of microbially treated PE films, with changes also observed in the profile of the PS films. The molecular weight of the PE pieces increased with incubation time, while the weight of PS pieces decreased. The buoyancy of PS pieces also changed due to the biofilm formation. The microbial diversity associated with two different microplastics (PP and PVC) increased over time when exposed to seawater [20]. Additionally, the microplastic biofilms exhibited contrasting microbial community structures in different marine environments. The surfaces of microplastics were enriched with bacteria capable of degrading hydrocarbons. Similar results were obtained in a mesocosm experiment [21] that examined the role of hydrocarbon‐degrading bacteria in breaking down PET. Significant differences in biofilm biodiversity were observed, with PET surfaces attracting distinct communities of hydrocarbon‐degrading bacteria. Major alterations in the surface chemistry and morphology of PET films have also been observed, which are mainly ascribed to the bacterial consortia enriched on tetradecane and diesel. Different changes have also been observed in a microcosm experiment involving the degradation of weathered PS films under simulated marine conditions [22]. The two tailored consortia efficiently reduced the weight of PS films. Fourier transform infrared (FTIR) spectrophotometry was used to detect alterations in the intensity of functional groups, along with signs of bio‐erosion on the PS surface. The results indicate that acclimated marine populations are capable of degrading weathered PS pieces.

Many other papers and reviews have reported similar results relating to the structural changes and fragmentation of plastic materials in natural environment. The consistent finding is that all degradation processes are slow and rarely lead to the complete destruction of plastic materials in natural waters.

## Microplastics in marine organisms

### Mussels

**Fig. 1:**
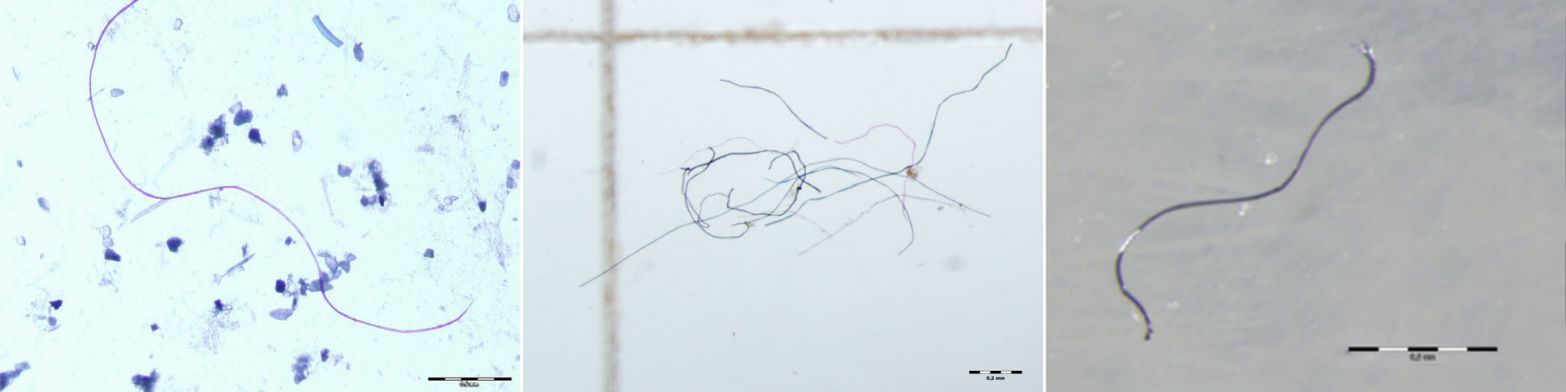
Fibers in sediment, fish (sea bass), and mussel (Mediterranean mussel) from the Gulf of Trieste (northern Adriatic). Photo M. Grego and O. Bajt, National institute of biology, Slovenia.

Mussels are filter feeding organisms that process large volumes of water (on average, 7–8 L of seawater per hour). As a consequence, they accumulate and concentrate many pollutants found in sea water. Mussels have a wide geographical distribution, which makes them ideal for extensive studies of coastal areas. In addition, they are sessile organisms that do not migrate, making sampling quite simple. This makes them good indicator organisms for seawater pollutants. As such, they have received attention in efforts to monitor microplastics in the marine environment (Fig. 1). Mussels, as well as other bivalve mollusks, are important organisms in marine food webs and can contribute to the transfer of microplastics to organisms at higher trophic levels [23].

A growing number of studies at various sites around the world have been conducted to monitor microplastic pollution using mussels. Phuong *et al*. [24] found eight different plastic materials in blue mussels (*Mytilus edulis*) from the French Atlantic coast, with PE and PP predominating. Most microplastics were fragments. On average, one particle per mussel was detected. A study of wild *M. edulis* in the coastal waters of the United Kingdom [25] revealed levels of six items per individual, of which micro‐FTIR spectroscopy revealed that only about 50% were microplastics. Rayon and cotton particles comprised the second most abundant group. In Norwegian waters, mussels from the relatively remote Barents Sea were compared to those from the highly urbanized Oslofjord [26]. Mussels were found to contain 13 different polymers, the most common of which were cellulose‐based polymers and small black rubbery particles that may have come from ‘road‐dust’. An average of 1.5 particles/individual were detected, with fibers accounting for 83% of particles identified. The sites studied did not differ in terms of the amounts of particles. However, site effects were detected in the amount of microplastics found in Mediterranean mussels (*M. galloprovincialis*) collected from coastal and offshore areas of the northern and central Adriatic Sea [27]. Mussels in coastal waters had microplastic loads about two times higher than those further offshore. Smaller particles (20–40 μm) were the most prevalent in both areas, while the most common polymer types were, in descending order, PE, PP, and PET, with smaller but equal amounts of PS, PA, and PVC. Much higher numbers of microplastic particles were detected in mussels in the Bizerte lagoon (northern Tunisia), which is exposed to a number of major anthropogenic pressures [28]. The average number of particles ranged from 2.6 to 12 items/mussel. The most common polymer in the mussels and seawater was PE, followed by PP. High levels of ingestion or deposition of particles were linked to areas that were highly polluted with fibers and PE. Filaments (fibers) were also the main microplastics in *M*. *galloprovincialis* surveyed in different Italian areas [29]. The average number of particles in individual mussels ranged from 3.0 to 12.4. No significant differences were found between cultivated and wild mussels, while cooking was found to reduce the presence of microplastics by up to 50%. Much lower numbers of particles were detected in the same species along the Turkish coasts [30], with an average of 0.69 items/mussel. Most particles were fragments, followed by fibers and a small portion of films. Twelve different polymers were detected, with PET, PP, and PE representing 80% of total microplastics. Almost half of *M. galloprovincialis* sampled in the northern Ionian Sea contained microplastics [31]. The largest portion of microplastics were fragments, and the main polymeric material was PE.

Results have also been reported for other mussel species. Microplastics in the ribbed mussel (*Aulacomya atra*) were investigated in an urban area in Patagonia, Argentina. On average, the mussels contained 0.3/g w.w. of microplastics with an average particle length of 1.25 mm, with fibers representing the dominant type [32]. About 96% of sampled mussels (*Limnoperna fortunei*) in the freshwater‐mixohaline tidal zone of the Río de la Plata estuary in Argentina contained microplastics [33]. Of the microplastics found, 90% were fibers. Microplastic levels were higher in mussels located closer to the main sewage discharges. Three mussel species (*M. galloprovincialis*, *Choromytilus meridionalis*, and *A. atra*) were analyzed along the coast of Cape Town, South Africa. Microplastics were recorded in 98% of mussels analyzed, with filaments being the most abundant. The study found a high average number of particles per individual mussel (4.27), with no significant differences between different mussel species. In the case of the Asian green mussel (*Perna viridis*) sampled along the southeastern coast of India, polystyrene was the only polymer identified [34]. Qu *et al*. [35] analyzed microplastics in *M*. *edulis* and *P. viridis* at 25 sites in the coastal waters of China to determine the relationship between the microplastics found in mussels and the surrounding waters. The authors found a quantitative correlation between microplastics in mussels and the surrounding waters, though mussels were more likely to ingest smaller particles.

### Fish

Much recent attention has been focused on the ingestion of microplastics by fish (Fig. 1) due to their importance in human nutrition and the growing prominence of farmed fish. Fish are also important organisms in marine food webs and play an important role in oceanic organic matter cycling. These studies have examined microplastic ingestion in wild and farmed fish in the field and in laboratory experiments around the world.

The Mediterranean Sea has been a particular focus of studies on microplastics and fish. The occurrence of microplastics in edible fish species (*Mullus barbatus* and *Merluccius merluccius*) has been measured in three different geographical subareas of the Mediterranean Sea [36]. Plastic fragments were detected in 23.3% of all fish, with fibers as the main source. The two species differ in the frequency of plastic ingestion, with microplastics twice as abundant in *M. merluccius*. The ingestion of anthropogenic particles was also studied in two areas of the western Mediterranean Sea [37]. Four fish species (*Trachurus mediterraneus*, *Sardina pilchardus*, *Engraulis encrasicolus,* and *Boops boops*) were included in the study. Anthropogenic particles were identified in the gastrointestinal tract of 28% of sampled fish. These particles were most frequently found in *T. mediterraneus*, while *E. encrasicolus* had the lowest percentage of affected individuals. Some differences between different study areas were also observed. Marine litter has been found in the stomach content of sardines (*S. pilchardus*) and anchovies (*E. encrasicolus*) in the central‐western Adriatic Sea [38]. Over 90% of individuals from both species contained marine litter as well as microplastics. Sardines contained a higher number of microplastics (4.63/individual) than anchovies (1.25/individual). These microplastics were predominantly comprised of five polymers, namely PP, PVC, polyacrylates, polytetrafluoroethylene (PTFE), and PE. These species were also investigated in the Gulf of Lyon [39]. There, microplastic debris was found in 12% of sardines and 11% of anchovies. Fibers were the only particles detected and PET was the main polymer identified, with smaller contributions of PE, PP, and PA.

Microplastic litter has also been identified in the benthic flatfish *Solea solea* from the northern and central Adriatic Sea [40]. Microplastics were recorded in 95% of sampled fish, with more than one microplastic item found in around 80% of the examined specimens. The most commonly found polymers were PVC, PP, PE, polyester, and polyamide, with fragments representing 72% of particles and fibers 28%. The polymer abundance exhibited some spatial variation. Microplastic ingestion by *B. boops* was studied in the Tyrrhenian and Ligurian Seas [41]. About 56% of gastrointestinal tracts contained microplastics. The most frequent materials were PE and PP, though some PS particles were also detected. The physical condition and sex appeared to have some impact on microplastic ingestion, with males ingesting more microplastics. *B. boops* was also used to assess microplastic ingestion at 20 sites in France, Italy, Spain, and Greece [42]. Microplastics were found in 46.8% of the sampled fish. Filaments were the predominant type, followed by fragments and films. The most common polymers were PE and PP, with PVC, nylon, and PET also present. The same species (*B. boops*) was sampled at three areas off the Catalan coast of Spain [43] with a similar portion of specimens found to have ingested microplastics (46%). Fragments and PP were the main type and polymer, respectively. The presence of microplastics and the particle types did display some geographical heterogeneity, mainly related to nearby coastal anthropogenic pressures. Sea bass (*Dicentrarchus labrax*), seabream (*Diplodus vulgaris*), and the flounder (*Platichthys flesus*) have been studied to assess the occurrence of microplastics in the waters of the Mondego estuary in Portugal [44]. Microplastics were found in 38% of all fish, with fibers representing the main type. Some interspecies differences were observed, with the levels of ingested microplastics significantly higher in *D. vulgaris*. The dominant polymers were PE, PP, and rayon.

Microplastic ingestion has also been studied in deep‐sea fishes. Mesopelagic fishes from the northwestern Atlantic exhibited a high ingestion rate of microplastics, with one study finding them in *Gonostoma denudatum* (100% of sampled fish), *Serrivomer beanii* (93%), and *Lampanyctus macdonaldi* (75%) [45]. The most frequent polymers were PE, methylcellulose, and nylon, with fibers representing the main particle type. Some differences between different pelagic and deep‐sea fishes have been reported in the northeastern Atlantic [46]. Pelagic species had significantly more microplastic than the deep‐water species. Fragments were the main shape recovered in both groups, but the prevailing material did differ between the two, as PP was more commonly found in deep‐sea fishes and PE was most abundant in pelagic fishes. The proportion of fish containing plastic items varied from 3.7% to 16.7% of individuals sampled, with an average of 9.49%. The ingestion of microplastics by deep‐sea fishes was studied in South China Sea [47]. All fish samples were contaminated with microplastics, with no differences between fishes from different depths. The largest portion of particles were under 1 mm, and the main particle type was film, followed by fiber and granule. Cellophane was the most common polymer found in all fish (56.8%), followed by PA (20.0%), PET (8.8%), and polyarylamide (PARA; 5.6%). Wang *et al*. [48] presented an analysis of microplastic uptake by 29 commercial fish species with different feeding habits and trophic levels from the Bohai Sea, China. Approximately 85.4% of all fish were found to have ingested microplastics. Fibrous particles predominated, with cellophane, PET, and PP representing the main polymer materials. Fish habitat is an important factor in microplastic ingestion, since benthivores had the highest abundance of microplastics and the highest diversity of materials. In contrast, no difference was found between fishes at different trophic levels in the food web. Significantly more microplastics were detected in six major wild fish species in Haizhou Bay, China, an important fish farm and mariculture area [49]. On average, 1.6–22.2 particles per individual were found in these fish species. The dominant particle type was fiber, and the predominant material was cellophane, followed by PP and PE. The gray mullet (*Mugil cephalus*) from the eastern coast of Hong Kong was found to have ingested microplastics in 60% of the wild fish sampled, while only 16.7% of mullets from fish farm had ingested microplastics [50]. The presence and characterization of microplastics were studied in six commercially important fish species, from different trophic levels, in central Chile [51]. The microplastics found in sampled fish were mainly comprised of fibers and polyester, with PE and PET as the prevalent polymers. Coastal species were found to have ingested microfibers with greater size and abundance than oceanic species. The ingestion of debris by the benthic foraging fish species *Acanthopagrus australis* (yellowfin bream), *M. cephalus,* and *Gerres subfasciatus* (silver biddy) has been quantified and characterized in Sydney Harbour, Australia [52]. About 53% of the ingested debris was microplastics. The most common polymers found were polyester, acrylic polyester, and rayon.

Overall, the literature demonstrates that microplastic pollution is widespread around the world. Even very remote and sensitive areas, like the Arctic, are affected by microplastic pollution. Two Arctic fishes, the demersal bigeye sculpin (*Triglops nybelini*) and the pelagic polar cod (*Boreogadus saida*), were collected off northeastern Greenland, with microplastics found in 34% of the former and 18% of the latter [53]. Polyester was the main polymer, followed by acrylate, PE, and PA.

The results presented in this section show that microplastics have become a ubiquitous problem among marine species of every size, trophic level, feeding behavior, distance from the coast, and depth habitat. They have been found in very remote areas, in the deep sea, and in the initial stages of marine organisms’ development (larvae and small fish). Due to the extensive number of publications in recent years, this review is limited to studies of mussels and fish. However, recent studies have presented results of microplastic ingestion by other marine organisms, including crustaceans, sea mammals, zooplankton, meiofauna, and seabirds.

## Effect on marine organisms

Marine animals are attracted to plastics due to their color, shape, and especially through algae that grow on plastic particles floating in sea water or settled on the sea bottom. Microplastics are quite easily ingested by marine organisms due to their small size. Once ingested, microplastics can cause different adverse effects on feeding behavior, reduction of predatory performance, reproduction, and energy budget, as well as inducing inflammatory responses, histological changes, DNA damage, cytotoxicity, physical damage, and mortality [23, 54]. Microplastic particles have a very high specific surface area, enabling the adsorption of different biomolecules that interact with biological systems, and, even more frequently, the adsorption of persistent organic pollutants. This provides a vector for the transportation of these pollutants by particles floating in seawater over long distances. After the ingestion by different animals, such pollutants can be leached from the particles and enter animal tissues, where they can disrupt normal biochemical processes in the cells [55]. Microplastic particles also represent a source of hormone‐disrupting compounds themselves due to additives that are added during the manufacturing process to achieve particular characteristics. Perhaps the most known additives are plasticizers (phthalates), bisphenol A, and polybrominated compounds (flame retardants), but many other are also used (e.g., pigments, biocides, and metals).

This section presents recent results on the impact of microplastics on mussels and fishes. These results are mostly from laboratory experiments, probably due to methodological issues.

The expression of biomarkers of oxidative stress (lipid peroxidations‐LPO, glutathione peroxidase‐GPx, acetylcholinesterase‐AChE, superoxide dismutase‐SOD) in Mediterranean mussels (*M. galloprovincialis*) was studied following their exposure to suspensions of microparticles, irregularly shaped PET fibers ranging in size from 5 to 3000 µm at concentrations of 0.1 g·L^−1^ [56]. Microplastics induced biochemical stress in mussels after 7 days of exposure, with a greater expression of biomarkers resulting from larger PET particles. The most sensitive enzymes were LPO and GPx. More studies have focused on the effect of adsorbed pollutants and leached pollutants from microplastic particles on mussels. *Mytilus galloprovincialis* has been used as a test organism to monitor the effect of low‐density polyethylene (LDPE) microparticles, both virgin and precontaminated with benzo[a]pyrene (BaP), a recognized cancerogenic polycyclic aromatic hydrocarbon [57]. Organisms were exposed for 4 weeks to LDPE at 10 mg·L^−1^ with particles ranging in size from 20 to 25 μm. The tissue localization of microplastics was histologically evaluated, chemical analyses of BaP were performed, and a wide battery of biomarkers were evaluated. Microplastics were localized in the hemolymph, gills, and especially the digestive tissues where a potential transfer of BaP from microplastics was also observed. Significant alterations were found in the immune system, while other effects were more limited. The weathering of microplastic particles significantly increased the weight of ingested particles [26]. Some structural changes to the gills and digestive gland were observed after the ingestion of PE particles, as well as necrosis in other tissues such as the mantle. The long‐term impact of microplastic exposure on two sediment‐dwelling bivalve species, *Ennucula tenuis* and *Abra nitida*, resulted in significant changes in energy reserves, while survival, condition index, or burrowing behavior was not affected [23]. The effects were linked to the size and quantity of particles, with more severe impacts attributed to larger particles and higher concentrations.

The excretion of microparticles by marine organisms is also an important process influencing the cycling, accumulation, trophic transfer, and toxicological effects in marine organisms. Different conditions influence the excretion of particles. Food availability prolongs the retention time of microparticles in mussels [58]. The lack of food causes rapid excretion of microplastics from mussels. The gut retention time is also related to the size of microparticles [59]. Smaller particles were generally excreted immediately, while the excretion of larger particles was much slower. The ability to rapidly eject smaller particles reduces oxidative stress in mussel organs [60].

The toxic effect of microplastics on fish has also been the focus of a number of studies. European sea bass (*D. labrax*) has been used to measure the effects of PVC and PE [61]. After 3 weeks of feeding with high concentrations of microparticles, a histological study revealed histological alterations in the liver and intestine. Ingestion of both PVC and PE particles slightly depressed the immune system, while PE microparticles alone resulted in a certain level of oxidative stress. Oxidative stress was induced by very small plastic microparticles (< 3 μm) in wild *Serranus scriba* caught along the Tunisian coast [62]. Microparticles were mainly found in the gastrointestinal tract and the musculature. Different polymers were detected in the fish tissues, including polyethylene‐vinyl‐acetate, HDPE, LDPE, PS, and PA. Long‐term exposure (90 days) of gilthead seabream (*Sparus aurata*) to LDPE microparticles increased the activities of antioxidant enzymes and caused a pro‐inflammatory response in gut, but after 30 days of depuration all the biomarkers analyzed tended to normalize, with the majority recovering values similar to those of the control group [63].

Microplastics induce toxic effects not only in adult fishes but also in the early stages of fish development. Pannetier *et al*. [64] presented results on the physiological and behavioral effects caused by fish consuming environmental microplastics, based on collections of individuals at different life stages from three islands near the North and South Pacific Gyres. Ingestion of microplastics by medaka larvae caused death, decreased head/body ratios, increased ethoxyresorufin‐O‐deethylase (EROD) activity and DNA breaks, and caused alterations to swimming behavior. EROD activity and DNA breaks were ascribed to the leached plastic additives and pollutants adsorbed on the plastic surface. Two‐month‐old juveniles did not exhibit any symptoms except an increase in DNA breaks. The toxicity of microplastics was related to polymer composition. Micro‐ and nanosized particles had adverse effects on goldfish larvae at higher concentrations, causing oxidative stress, damage to intestine, liver and gill tissues, increased heart rate, and inhibited growth and movement [65]. These toxic effects are not only the result of microplastic ingestion, but also due to the agglomeration of particles on the surface of animals, especially on the surface of the chorion of marine fish larvae [66]. Different pollutants adsorbed on microparticles decreased embryonic survival and prevented hatching, while also causing reduced growth, increased developmental anomalies, and abnormal behavior.

## Conclusions and perspectives

This overview of recent papers on the problem of microplastics in the marine environment, with a focus on mussels and fishes, confirmed the rapid increase of published results in this research area. Microplastic pollution is a very widespread problem, with microparticles of artificial polymers found in almost all regions of the world, including very remote areas with no important pollution sources. Microplastics have been identified in different mussel and fish species, at least in low concentrations. However, in many cases the proportion of individuals found to have ingested plastic particles exceeds 50% and sometimes approaches 100%. This problem is becoming more and more widespread, since the degradation of plastic material in the natural environment is a very slow process, primarily driven by mechanical and photochemical processes, though biodegradation also plays a role. Unfortunately, these processes very rarely lead to complete short‐term degradation of different plastic materials. Many studies also emphasize the adverse toxic effects on marine organisms after the ingestion of microplastics. Fortunately, many organisms show the ability to excrete microparticles, reducing the impact on their health, but not the global problem of microplastic pollution.

While the results, presented in this paper, do provide an overview of this problem, they are often difficult to compare. Different sampling and analytical approaches are used, as well as a wide variety of species. There is a pressing need to unify the protocols involved in such sampling and analyses, at least on a regional scale. Similar approaches may be drawn from studies of other pollutants in order to define analytical procedures, indicator organisms, and testing conditions, expression of results, among other considerations. A special issue is the study of toxicological effects of microplastics on marine organisms. Because of methodological issues, this is usually tested in laboratory conditions, which are quite often very different from the real field conditions (in terms of the concentration and mixtures of microplastics, temperature, influence of waves, currents, exposure time, health and nutrition conditions, age, size, and other considerations). I believe that distinguishing between the effects of particles with adsorbed pollutants and the presence of such pollutants in natural ecosystems, as a consequence of pollution, is also an important issue. Marine organisms come in contact with them in water and sediments when they feed and breathe. These problems should be addressed in the future.

## Conflict of interest

The authors declare no conflict of interest.

## Author contributions

OB wrote the manuscript and prepared the figure and tables.

## Data Availability

This is a review paper, all data were obtained from the Scopus and WoS databases, as it is written in the text.
